# Characterization of particle size segregation and heterogeneity along the slopes of a waste rock pile using image analysis

**DOI:** 10.1007/s12665-023-11229-y

**Published:** 2023-11-15

**Authors:** Peiyong Qiu, Thomas Pabst

**Affiliations:** 1 Department of Civil, Geological, and Mining Engineering, Polytechnique Montréal, Montréal, Québec Canada; 2 Research Institute On Mines and Environment (RIME), Montréal, Québec Canada; 3 Norwegian Geotechnical Institute, Oslo, Norway

**Keywords:** Mine waste rock, Segregation, Lateral heterogeneity, Image analysis

## Abstract

Large amounts of waste rock are produced during mining operations and often disposed of in large piles. Particle size segregation usually occurs during waste rock disposal, which can lead to high variations of particle size distribution (PSD) along the pile slope, increasing the risk for hydrogeotechnical instabilities. Determining segregation in situ is, therefore, critical to implement control measures and optimize deposition plans. However, characterizing PSD at field scale remains challenging because of the large dimensions of the pile, the instability of the blocks and the steep slopes. In this study, images, covering a 1400 m wide and 10 m high section of a waste rock pile, were taken and analyzed using image analysis to characterize segregation along the slope of the pile. PSD curves in different sections along the slope were determined and the segregation degree and characteristic diameters (e.g., *D*_10_, *D*_50_, *D*_80,_ *D*_95_) were quantitatively compared. Results allowed to quantify segregation along the vertical direction of the pile, showing that segregation degree increased from − 0.77 ± 0.39 in the top (finer zone) to + 0.4 ± 0.14 in the bottom (coarser zone). Significant lateral heterogeneity was also observed with maximum diameters varying between 80 and 180 cm in the bottom section. Such segregation and lateral heterogeneity could induce significant variations of waste rock properties, with, for example, hydraulic conductivities varying by more than 2 orders of magnitude within the pile.

## Introduction

Large amounts of waste rock are generated in mining operations and then disposed of in large piles that can exceed hundreds of metres in height and several square kilometers in area (Mclemore et al. 2009; Aubertin 2013; Bar et al. 2020). The particle size distribution (PSD) of waste rock ranges from clayey particles to metre-sized blocks (Morin et al. 1991; James et al. 2013). Waste rock piles are usually constructed in benches which are typically between 10 and 25 m high. Waste rock is repeatedly dumped down the slopes by haul trucks with either end-dumping method or push-dump method (Aubertin 2013; Amos et al. 2015; Hajizadeh Namaghi et al. 2015).

These construction methods usually lead to significant segregation: larger (i.e., heavier with more momentum) particles tend to move down the slope and create a coarse zone at the base of the benches, while finer particles are more easily blocked and tend to remain closer to the crest and the deposition point (Herasymuik et al. 2006; Van Staden et al. 2018). Segregation is a complicated process involving different mechanisms, such as inertial segregation (Goyal et al. 2006), sieving effect (Mosby et al. 1996) and percolation (Khola et al. 2016). Segregation is also affected by material properties, such as the original PSD particle shape and roughness (Alizadeh et al. 2017). Continuous traffic of construction equipment can also create compacted layers of crushed material at the surface of the benches (Fala et al. 2005; Bao et al. 2020; Maknoon et al. 2021). Fine and coarse-grained inclined layers, therefore, alternate in the pile profile as the waste rock pile advances (Azam et al. 2007; Anterrieu et al. 2010).

Consequently, waste rock piles are highly heterogeneous structures (Anterrieu et al. 2010; Lahmira et al. 2016; Van Staden et al. 2019). These heterogeneities can directly affect the spatial distribution of geotechnical and hydrogeological properties (e.g., shear strength, hydraulic conductivity) and cause preferential flow, thus increasing the risk for both geochemical and geotechnical instabilities (Lahmira et al. 2016; Martin et al. 2019; Bréard Lanoix et al. 2020 et al. 2020).

Much effort was, therefore, put to characterize particle size distribution of waste rock in piles but also rockfill in dams, both in the field (Essayad 2021; Wilson et al. 2022) and in the laboratory (Shepherd 1989; Ayres et al. 2006; Gupta 2016). Most of the research, however, focused on small scales with particles usually smaller than 0.5 m (Cash 2014; Barsi 2017). Sieve analysis (ASTM D6913) for the coarse fractions (> 0.075 mm), combined with wet sieving (ASTM C117–17 2017) and hydrometer tests (ASTM D7928–17 2017) for fine fractions (< 0.075 mm), are indeed traditionally the most used approaches to characterize the PSD of waste rock (and other mine wastes) (Bao et al. 2020; Hao and Pabst 2022; Essayad 2021).

Sieve analysis can be accurate to determine the PSD of samples, but limited sieve sizes and the practical difficulties to obtain and transport large samples may affect the representativeness of the samples, which may not reflect the whole particle sizes in field conditions (Hawley et al. 2017). Sampling and field characterization along the slope of a waste rock pile is also dangerous because of the large dimensions of the pile, the instability of the blocks and the steep slopes (Raymond et al. 2021). Characterizing segregation of waste rock at large scale, therefore, remains a challenge.

Recently, using a drone, combined with image analysis, was proposed to determine waste rock PSD in the field (Dipova 2017; Zhang et al. 2017). Image analysis is widely applied in different industries and research areas, including particle mixing in rotary drums (Liu et al. 2015), soil crack analysis (An et al. 2020; Zhang et al. 2021; Meng et al. 2022), rock fragmentation (Andriani et al. 2002; Lawal 2021) and mining engineering (Fernlund 2005; Al-Thyabat et al. 2006; Ko et al. 2011). The approach usually consists in automatically extracting particle information, such as particle shape, area and axis lengths, but the main challenge for applications to waste rock characterization is that only two dimensions of each particle can be obtained from the 2D images, with the third dimension being hidden (Fernlund et al. 2007; Lee et al. 2007; Zhang et al. 2012).

In this study, waste rock segregation was investigated using image analysis. A total of 87 drone images, covering a 1400 m wide and 10 m high bench, were taken on the waste rock pile of Canadian Malartic mine, a surface gold mine located in Quebec, Canada. 42 images were selected to analyze the waste rock in these images. Waste rock segregation was characterized based on segregation degree and characteristic diameters, including *D*_10_ to *D*_95_. The lateral heterogeneity of waste rock was also investigated by analyzing the variability of the PSD curves and characteristic diameters (i.e., *D*_10_, *D*_50_ and *D*_95_) along the lateral direction of the slope.

## Methodology

### Digital image acquisition

**Fig. 1:**
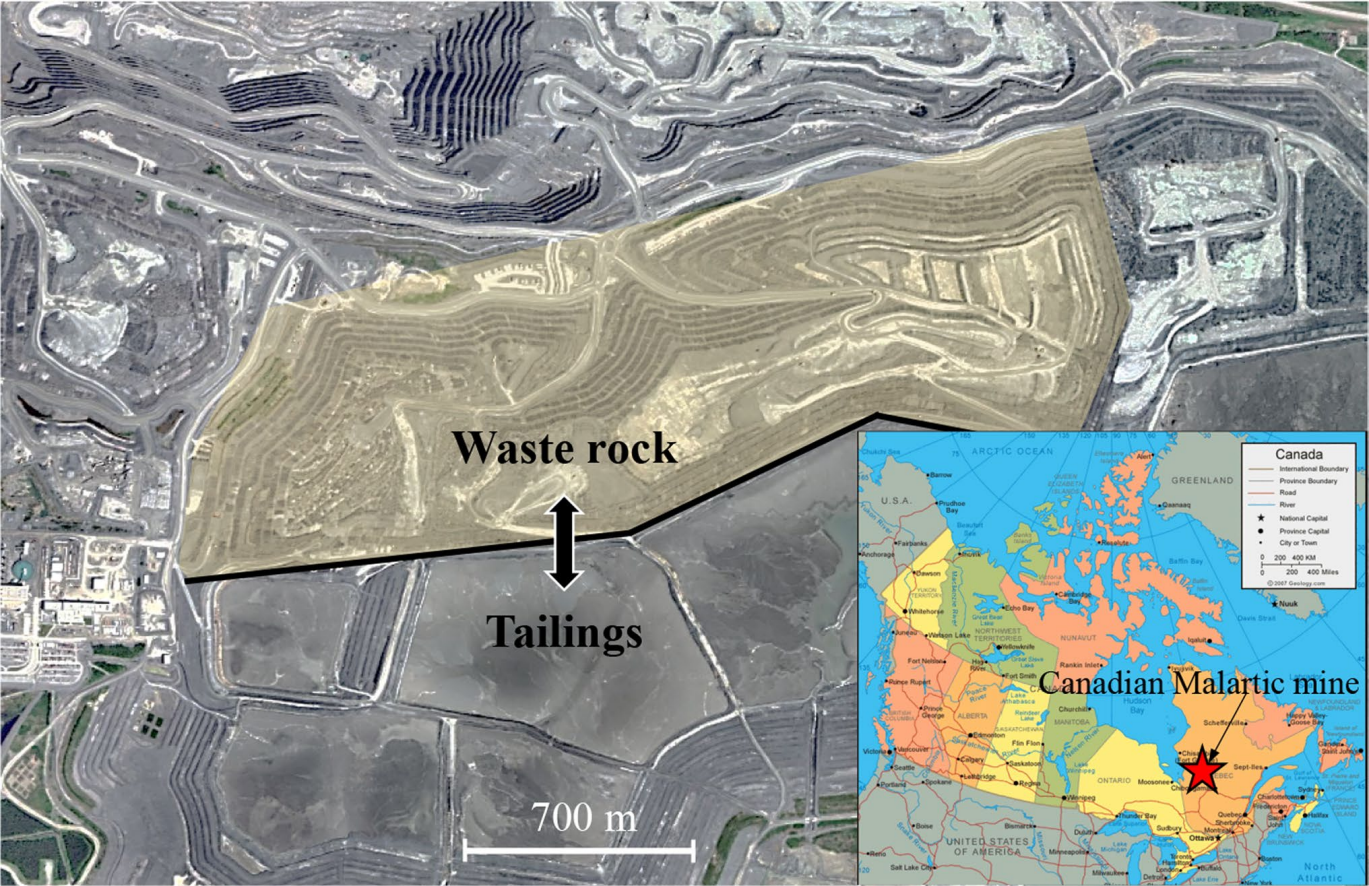
Waste rock pile and tailings storage facility at Canadian Malartic mine. The measured slope is located in the yellow area (Photo from Google Earth 2023). Location of the Canadian Malartic mine site is shown in the bottom right corner (Photo from Government of Canada)

This study was carried out on the waste rock pile of Canadian Malartic mine, which is an open-pit mine located within the Municipality of Malartic, approximately 25 km west of Val-d’Or and 80 km east of Rouyn-Noranda in Quebec, Canada (Fig. 1). The latest mine production schedule plans to feed the mill from the open pits and stockpiles at a nominal rate of 57,000 tons per day (Lehouiller et al. 2020). An estimated total of 450 Mt of waste is placed on the waste rock pile, at an in-situ compacted density of 1.96 t/m^3^, representing a storage volume of 230 Mm^3^. The mineral compositions of studied waste rock mainly include 25% quartz, 38% albite, 11% muscovite, 7% chlorite, 6% corundum, and 6% diopside (Hao and Pabst 2021). The waste rock pile is constructed in 10 m benches with 11.5 m terraces between benches for an overall slope angle of 21.8°. The slope angle of each bench is around 37° (Gervais et al. 2014). The total height of the waste rock pile reaches 100 m in most sections (Lehouiller et al. 2020). A total of 87 photos were taken using a camera installed on a drone flying parallelly to the slope of the pile. The camera was oriented orthogonally to the slope to avoid lateral distortion. The drone moved horizontally along the bench at a constant elevation of 50 m above the bottom of the slope. An accumulated around 4000 m long slope was photographed with a resolution of 5280 × 2970 pixels for each image. A 0.5 m × 0.5 m reference target was placed on the slope surface for size calibration. The pixel conversion factor was 1.2 cm. The top and bottom edges of each bench (i.e., compacted zones between benches) could be observed on each image and clearly delimited the investigated slope. Finally, a total of 42 independent photos (i.e., with no overlap) covering each around 30 m wide and 10 m high zone were selected for analysis based on the quality, brightness and resolutions of the images.

### Image processing

**Fig. 2:**
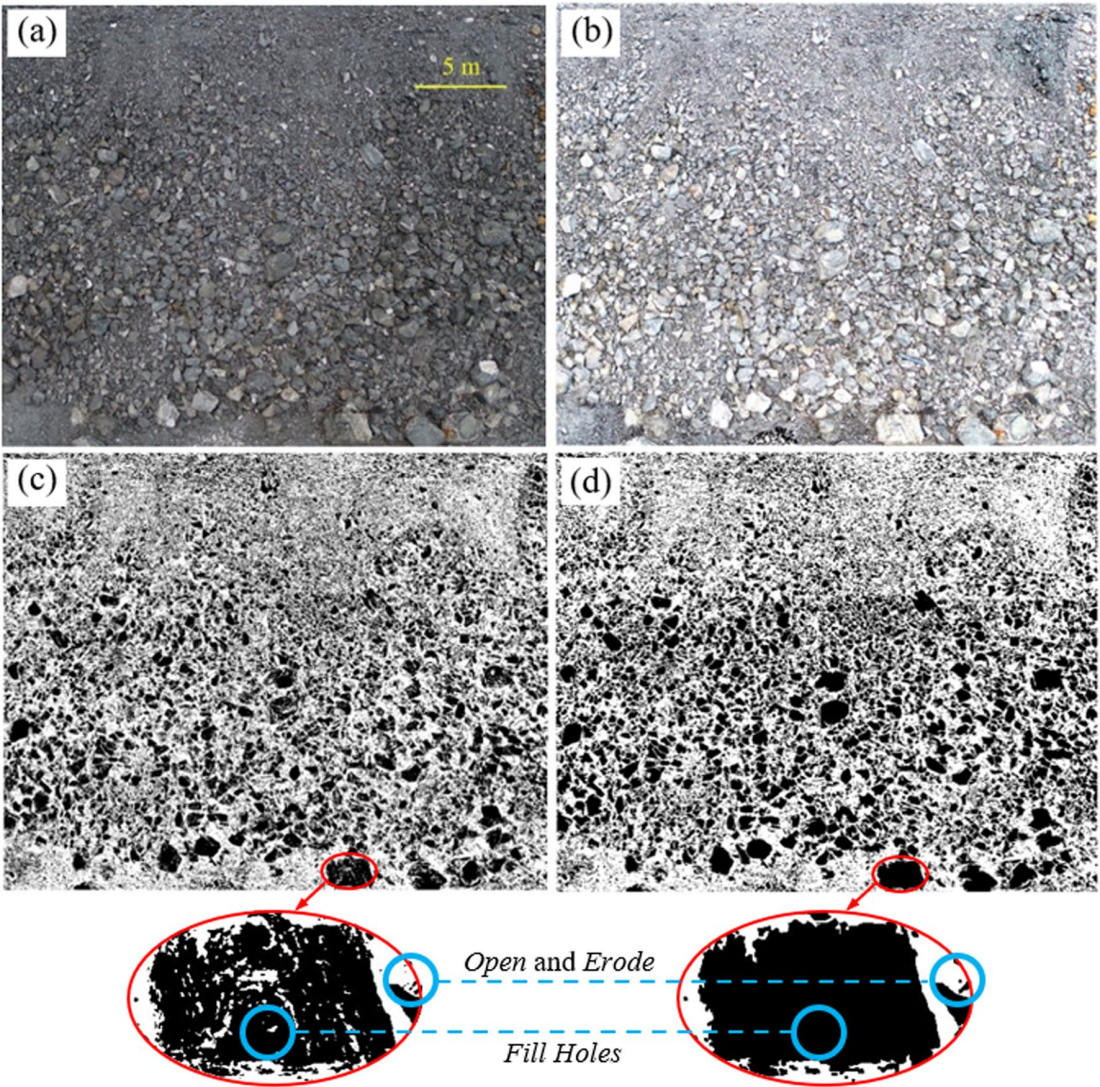
Processes of image analysis for waste rock along the slope. **a** Original waste rock on the slope surface of waste rock pile. **b** Pre-processing to increase the brightness and contract of the image. **c** Binary conversion. Black colour represented waste rock particles. **d** Final image after processing with functions such as *Open, Erode* and *Fill Holes* to improve the image quality (see text for details)

Waste rock particles were analyzed using ImageJ, an open-source program for image processing (Abràmoff et al. 2004; Ferreira and Rasband 2012). Original images (Fig. 2a) could not directly be processed for particle size distribution because of the difficulty to differentiate particles and voids. Indeed, waste rock exhibited colour differences because of differences in mineral composition and shades. Images were, therefore, processed using multiple sequential steps, including pre-processing, binary conversion, processes on binary image and particle analysis. These different steps are briefly presented and illustrated below.

*Pre-processing*: The original RGB colour images were first pre-processed using filtering, colour adjustment, brightness, and contrast adjustments to increase the brightness and enhance particle boundaries (Fig. 2b).

*Binary conversion*: Pre-processed images were converted to 8-bit grayscale images in which the gray level of the image ranged between 0 and 255 (0 representing black colour and 255 representing white colour). Thresholding was conducted to set lower and upper gray levels to distinguish particles from the background (Fig. 2c). The black areas represented waste rock particles in binary images. The white areas represented voids between particles, as well as particles below 2 cm diameter which were not considered in the analysis (more details below). The brightness and the arrangement of particles were not uniform between images, although they exhibited the same color system in general. As a consequence, the thresholding values also slightly varied between the images. A systematic approach was, therefore, used and consisted in adjusting the thresholding, so that the edge of large particles (which could be easily identified in the images) was clearly observed.

*Processes on binary image*: Binary images usually exhibited different defects, such as grains from the background of the image, or blurry boundaries between particles (Fig. 2d). Functional processes included in ImageJ, such as *Erode, Dilate, Open, Close, Fill Holes*, were used to remove this noise and make the transition between particles clearer. For example, two particles in contact might be detected as one particle in binary conversion process, so *Erode* function was used to remove pixels from the particle edges and separate the two particles. *Dilate* provided the opposite function to *Erode* by adding pixels, so that the edge of the particles could be more precisely determined. *Open* function smoothed objects and removed isolated pixels which were smaller than 1.2 cm and not considered in this study. *Close*, followed by *Erode*, performed a dilation operation to smooth particles and fill small holes by adding pixels. *Fill Holes* was similar to function *Erode* and was used to fill small holes inside particles.

*Particle analysis*: Processed binary images were finally used to extract particle information, including surface area, shape and axis lengths. The area of every individual particle was estimated from its pixels. The minimum detectable area in ImageJ was 25 cm^2^ (corresponding approximately to a diameter of 2 cm) and smaller particles were ignored to reduce the influence of noise and resolution. Ellipses were used to fit particles in binary images and the primary and secondary axes of ellipses were obtained. ImageJ provided a list of each individual particle information, including its area and primary and secondary axes lengths.

### Particle shape and diameter

The mass and diameter of particles are the two main properties used to determine the PSD curve. The mass of each waste rock particle was calculated by estimating its volume multiplied by its specific gravity (Gs = 2760 kg/m^3^ based on laboratory measurements following ASTM C127-15 2015). However, particle volume can be difficult to estimate from 2D images and various shape models, such as the sphere model (Zhang et al. 2017) and ellipsoid model (Podczeck 1997), are, therefore, typically used to convert 2D areas to 3D volumes. Ellipsoid model was successfully used in previous studies to determine the PSD curve of coarse-grained materials using image analysis (Kumara et al. 2012; Dipova 2017), and was, therefore, also used in this study.

**Fig. 3:**
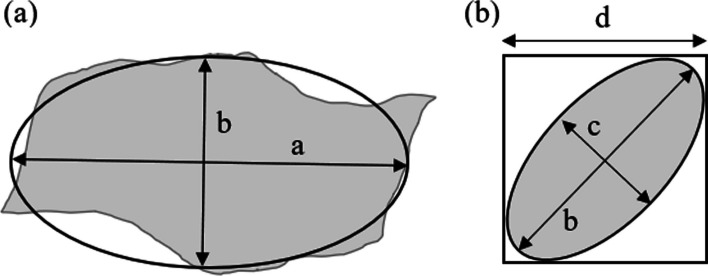
Dimensions of the ellipsoid model. **a** Projected area of a particle in image analysis; **b** particle passing through a square sieve. Lengths a and b are the primary (long) and secondary (medial) axes of the ellipsoid determined in image analysis. Length c represents the short axis of the best fitted ellipsoid. Length d is the square sieve aperture in sieve analysis

Ellipsoid particles are represented by a long (a; Fig. 3a), a medial (b; Fig. 3a, b) and a short axis (c; Fig. 3c). The volume (V) of an ellipsoid is then calculated as (Kumara et al. 2012)

1 $$V = \frac{4\pi }{3} \times \frac{a}{2} \times \frac{b}{2} \times \frac{c}{2}.$$

Particle diameter, in the sense of PSD analyses, typically corresponds to the size of the square sieve aperture (d in Fig. 3b) and can be smaller than the medial axis (b in Fig. 3). In other words, waste rock retained in a specific sieve is often slightly larger than its diameter (d). Therefore, an equivalent particle diameter (*D*_e_ = *d*) was introduced and was calculated based on the medial (b) and short axes (c) of waste rock in images (Kumara et al. 2011; Dipova 2017). The detailed derivation process can be found in Ohm et al. (2013). The equivalent particle diameter (*D*_e_) can be expressed as

2 $$D_{{\text{e}}} = \sqrt {\left( {b^{2} + c^{2} } \right)/2} .$$

Lengths *a* and *b* were obtained directly from image analysis (see above), but length *c* was hidden in the out of plane dimension. A shape factor *λ* (= *c*/*b*) was introduced (Podczeck 1997; Đuriš et al. 2016). Particles from the same source are considered to exhibit constant shape characteristics, i.e., *λ* is constant and independent of particle size (Mora et al. 1998; Dipova et al. 2017).

**Fig. 4:**
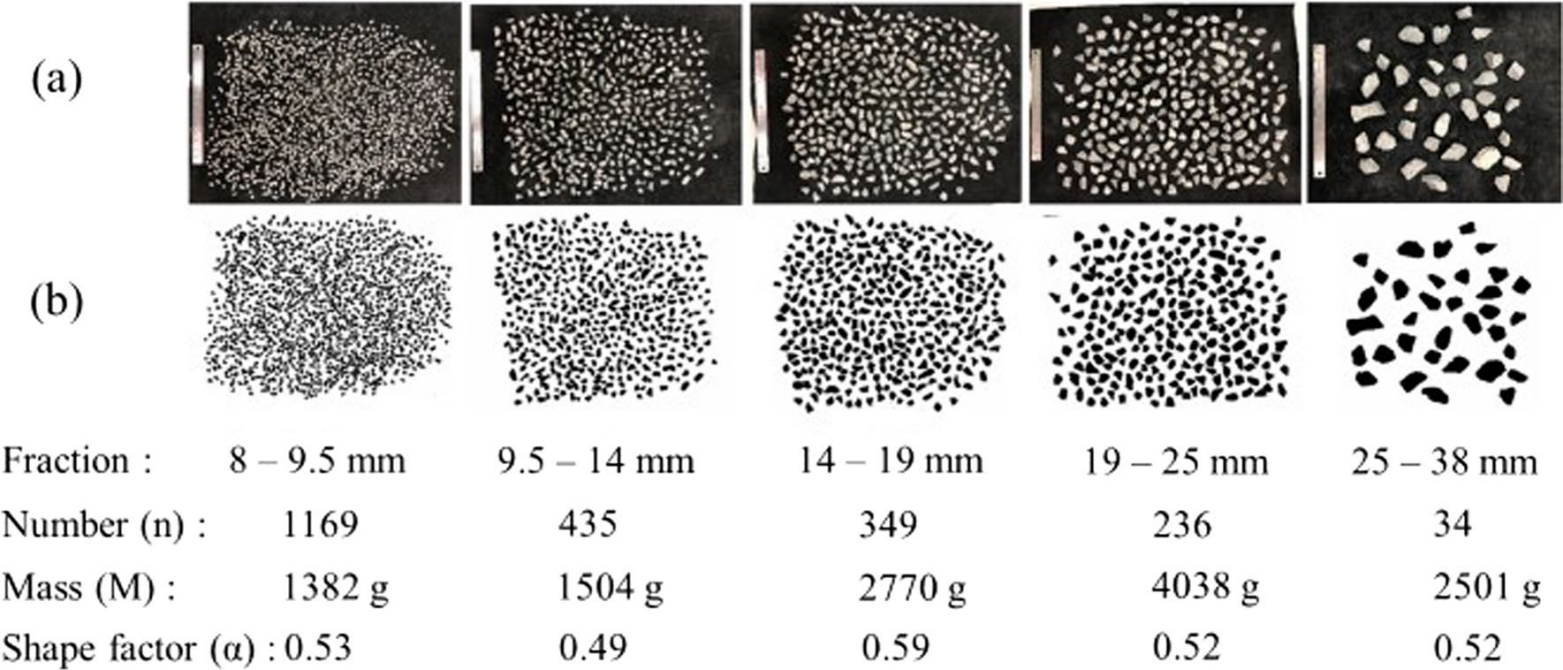
Determination of the shape factors with different size fractions. **a** Original images of waste rock with size reference (0.3 m long). **b** Binary images used for image analysis. The mass (M) of each fraction was measured using a balance in the laboratory

The shape factor $\alpha$ for the investigated waste rock was, therefore, calibrated in the laboratory by comparing sieve analysis and image analysis conducted on samples collected from the same pile at Canadian Malartic mine. Around 1 ton of waste rock was sampled from Canadian Malartic mine. The original waste rock was collected before the deposition, and particles greater than 5 cm were removed. Samples were sent to the laboratory in barrels, then dried, sieved and homogenized (ASTM C136, 2019 and D6323, 2019), before being stored in buckets by fractions until their characterization and use in the tests (see below). Five fractions ranging between 8 and 38 mm were prepared, spread on a black geotextile (no contacts) and photographed (Fig. 4). Images were taken vertically at a height of approximately 1 m, and a 0.3 m long ruler was used for size calibration (pixel conversion factor was 0.17 mm). The projected area of each particle was analyzed, and the long (a) and medial (b) axis were determined using ImageJ and the best fitting ellipsoid model, following the same procedure as described above for field image analysis.

The mass (M) of each waste rock fraction was first measured in the laboratory using a balance. The mass of every particle was calculated by multiplying its volume estimated using the ellipsoid model (Eq. 1) by its specific gravity. The short axis c in the ellipsoid model was expressed as *λb*. The mass of each waste rock fraction was then estimated by summing the mass of all the particles, and as a function of the shape factor λ. Thus, λ in each fraction could be calculated using Eq. 3. The average shape factor of the five fractions was 0.53 (Fig. 4), and was used to estimate the short axis c of waste rock particles in the field:

3 $$\lambda = \frac{M}{{\beta \times \mathop \sum \nolimits_{i = 1}^{n} \frac{4\pi }{3} \times \frac{{a_{i} }}{2} \times \frac{{b_{i} }}{2} \times \frac{{b_{i} }}{2}}}$$

where *M* is the mass of one waste rock fraction measured using a balance in the laboratory; ${\upbeta }$ is the specific gravity of waste rock particles.

### Segregation characterization

**Fig. 5:**
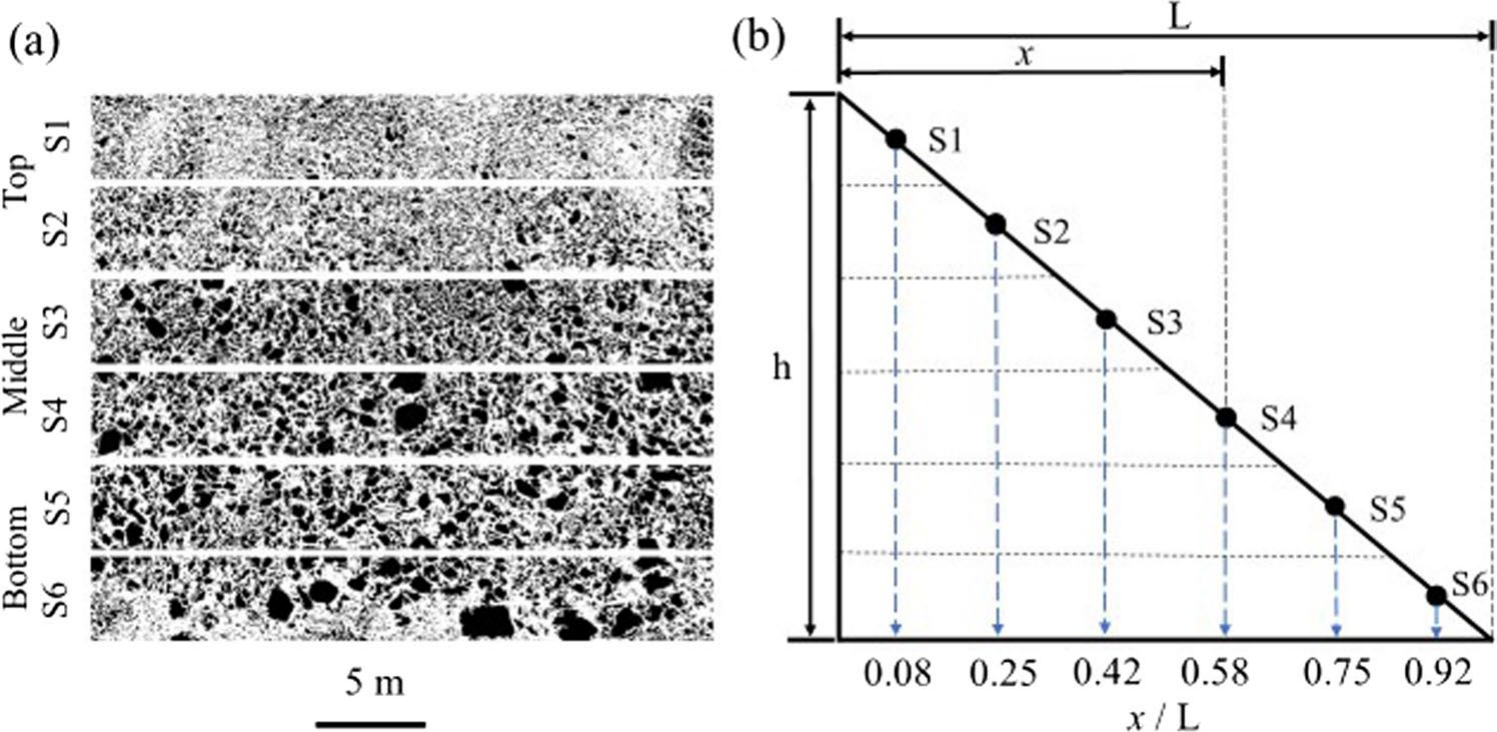
**a** Sectional binary images of waste rock with the slope divided into six sections (S1–S6). **b** Characterization of the six sections along the slope.* x*/L [–] represents the position of each section with *x* [L] the horizontal distance of the section center to the deposition point and L [L] the horizontal length of the slope. h [L] represents the height of the bench

Each of the 42 images was divided into 6 vertical sections (Fig. 5a) and PSD curves of each section were determined based on the ellipsoid model using a shape factor *a* = 0.53. The PSD curve of the original waste rock (before deposition and segregation) was determined from the image analysis of the entire slope in each image. This so-called original PSD curve was used to evaluate the waste rock segregation along the slope in each image. The characteristic diameters *D*_10_, *D*_15_, *D*_30_, *D*_50_, *D*_60_, *D*_80_, *D*_95_ (i.e., the particle diameters corresponding to 10%, 15%, 30%, 50%, 60%, 80%, and 95% passing, respectively), the coefficient of uniformity C_U_ (C_U_ = *D*_60_/*D*_10_) and the coefficient of curvature *C*_C_ (*C*_C_ = (*D*_30_)^2^/(*D*_10_ × *D*_60_)) of each PSD curve were determined. Characteristic diameters (*D*_10_, *D*_50_, *D*_80_, *D*_95_) in different segregated zones were used to characterize the segregation of waste rock in the field (Blight 2010; Zhang et al. 2017; Dalcé et al. 2019). The increase of these characteristic diameters along the slope showed that particles tended to become coarser.

Statistical analyses were conducted using Gauss distribution model. The mean and standard deviation of the passing percentage were obtained for each section and for the whole slope. Then, the mean and median PSD curves, and the PSD curves with passing percentage less than one standard deviation (σ) of the mean (68% of the data set), and PSD curves with passing percentage less than two standard deviations of the mean (95% of the data set) were obtained for each section.

The segregation of waste rock along the slope was characterized using segregation degree and relative particle diameters. The segregation degree (χ, Fig. 5) can well indicate the segregation state of waste rock in each section using the corresponding PSD curve. Relative particle diameter *D*_10_/*D*_10_ʹ (also *D*_50_/*D*_50_ʹ, *D*_95_/*D*_95_ʹ) was defined as the ratio between *D*_10_ in each section and *D*_10_ʹ in the original PSD curve in each image. The center of each section along the slope was characterized in Fig. 5b.

The segregation degree of waste rock (*χ*, Sutherland 2002) in each section was determined as

4 $$\chi = \frac{{\log d_{{{\text{tested}}}} - \log d_{0} }}{{\log d_{{{\text{CQ}}}} - \log d_{0} }}$$

where ${ }\log d_{0}$ is the logarithmic mean particle size of the original material before disposal, d was in cm in this study; $\log d_{{{\text{tested}}}}$ is the logarithmic mean particle size of waste rock in the local sections ((i.e., sections 1–6)); $\log d_{{{\text{CQ}}}}$ is the logarithmic mean particle size of coarsest quartile. The coarsest quartile represented the fraction of particles larger than the diameter *D*_75_ of the original gradation (Westland 1988; Kenney et al. 1993). Logarithmic mean particle size ($\log d$) is often considered a representative parameter for delineating the PSD curve, and is calculated as (Kenney et al. 1993)

5 $$\log d = \mathop \sum \limits_{i = 1}^{n} \left( {P_{i} - P_{i - 1} } \right)\log \sqrt {D_{i} \cdot D_{i - 1} }$$

where $D_{i}$ and $D_{i - 1}$ [L] are consecutive particle diameters corresponding to passing $P_{i}$ and $P_{i - 1}$.

## Results

### Segregation along the slope

**Fig. 6:**
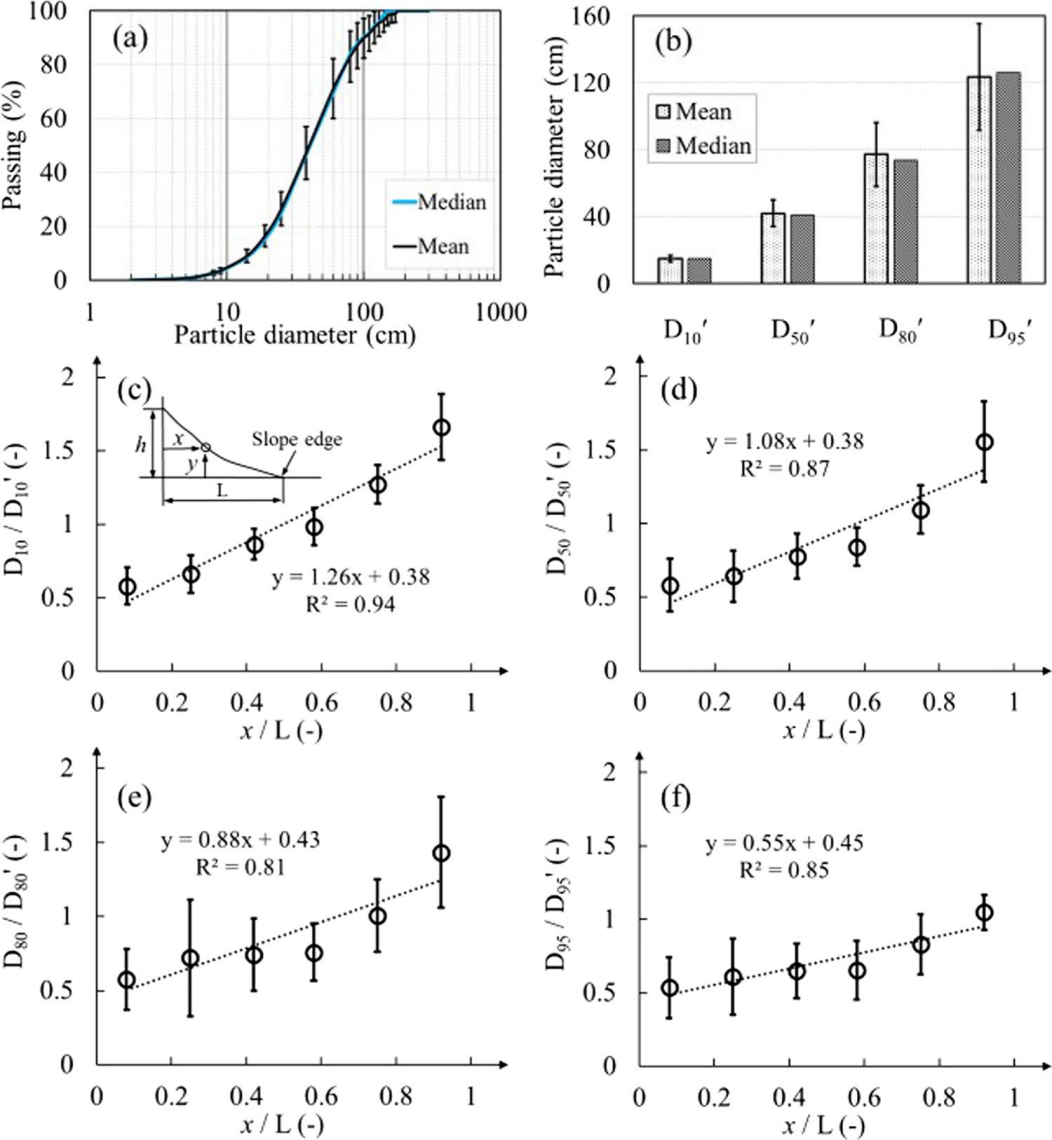
Segregation characteristics of waste rock in the six sections of the slope. **a** Measured median and mean PSD curves of the whole slope. **b** Measured characteristic diameters of the original PSD curves. The mean and median were obtained from the 42 PSDs. Measured **c** *D*_10_/*D*_10_ʹ, **d** *D*_50_/*D*_50_ʹ, **e** *D*_80_/*D*_80_ʹ and **f** *D*_95_/*D*_95_ʹ as functions of the section positions. Relative particle diameter D_10_/D_10_ʹ (also *D*_50_/*D*_50_ʹ, *D*_95_/*D*_95_ʹ) was defined as the ratio between D_10_ in each section and *D*_10_ʹ of the original PSD curve in each image. *x*/*L* [–] represents the position of each section with *x* [L] the horizontal distance of the section center to the deposition point and L [L] the horizontal length of the slope. Error bars represent standard deviation

A total of 42 original PSD curves were obtained from the 42 images. The maximum particle diameter was 180 cm among the 42 images. The original waste rock in each image was characterized by *D*_10_ʹ, *D*_50_ʹ, *D*_80_ʹ and *D*_95_ʹ (Fig. 6b). The maximum and minimum *D*_95_ʹ were 175 cm and 69 cm, with a mean and median of 123 cm and 126 cm. The maximum and minimum *D*_10_ʹ were 19 cm and 12 cm, with the same mean and median of 15 cm. The mean and median PSD curves were obtained from the 42 original PSD curves (Fig. 6a). The median and mean PSD curves exhibited the same *C*_U_ (= 3.4) and *C*_C_ (= 1.04). Difference between mean and median PSD curves mainly concentrated around particles larger than 130 cm, which only accounted for 4% of the total waste rock. Particles larger than 100 cm accounted for 10% of the total waste rock and fractions between 19 and 80 cm accounted for around 68%. Waste rock with diameters ranging between D_40_ and D_80_ exhibited the highest level of variability, with standard deviation around 10%, but otherwise results were similar (standard deviation < 8%).

Significant segregation was observed from the top to the bottom of the slope, i.e., from Sections 1–6 (Fig. 6c–f). For example, *D*_10_/*D*_10_ʹ increased from 0.58 ± 0.13 in Section 1 (top section, *x*/*L* = 0.08) to 1.66 ± 0.23 in Section 6 (bottom section, *x*/*L* = 0.92), i.e., an increase by 186%. *D*_50_/*D*_50_ʹ increased from 0.58 ± 0.18 in Section 1 56 ± 0.23 in Section 6, i.e., an increase by 168%. Similar trend was also observed from *D*_80_/*D*_80_ʹ and *D*_95_/*D*_95_ʹ, which increased by about 149% and 95%, respectively, from Sections 1–6. The increase rate of relative particle diameters decreased from 1.26 for *D*_10_/*D*_10_ʹ to 0.55 for *D*_95_/*D*_95_ʹ, thus indicating that smaller particles were more sensitive to segregation (Fig. 6c–f).

**Fig. 7:**
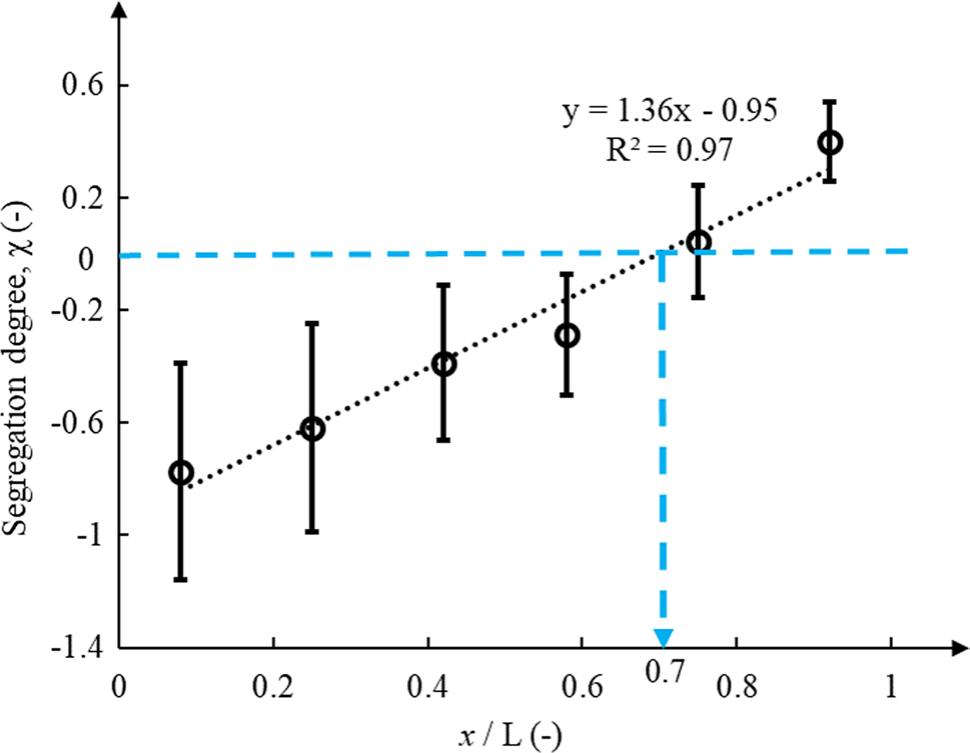
Segregation degree along the slope. Particles were finer than the original (i.e., *X* < 0; blue dashed line) when *x*/*L* < 0.7 and coarser than the original (i.e., *X* > 0) when *x*/*L* > 0.7. Error bars represent standard deviation

Segregation degree (χ) of waste rock along the slope also indicated significant segregation. Segregation degree was – 0.77 ± 0.39 in sections 1 and increased to – 0.29 ± 0.21 in sections 2 (*x*/*L* = 0.58), indicating that waste rock in sections 1 was significantly finer (+ 166%) than in sections 2 (Fig. 7). In addition, the segregation degree was smaller than 0 when *x*/*L* < 0.7 thus confirming that waste rock was finer than the original PSD in the top two-third of the slope. The segregation degree in Sect. 6 was significantly greater and around + 0.4 ± 0.14 and waste rock in the bottom of the slope was coarser than the original waste rock, but also coarser than waste rock in sections 1–6. Finally, the standard deviation of the segregation degree decreased from 0.39 in sections 1 to 0.14 in Section 6. Waste rock in the top sections was, indeed, significantly finer than the original waste rock and a few additional or fewer large particles would, therefore, strongly affect the PSD curve.

### Lateral heterogeneity

The 42 PSD curves analyzed above represented the PSD of waste rock on the surface of the pile slope, covering a lateral distance of 1400 m. As mentioned above, there was no overlap between the PSD curves so the data set was used to evaluate waste rock lateral variability. It was assumed, based on information received from the mine, that the geology of the rock was sensibly similar, and that the deposition method was the same (i.e., end dumping in that case).

**Fig. 8:**
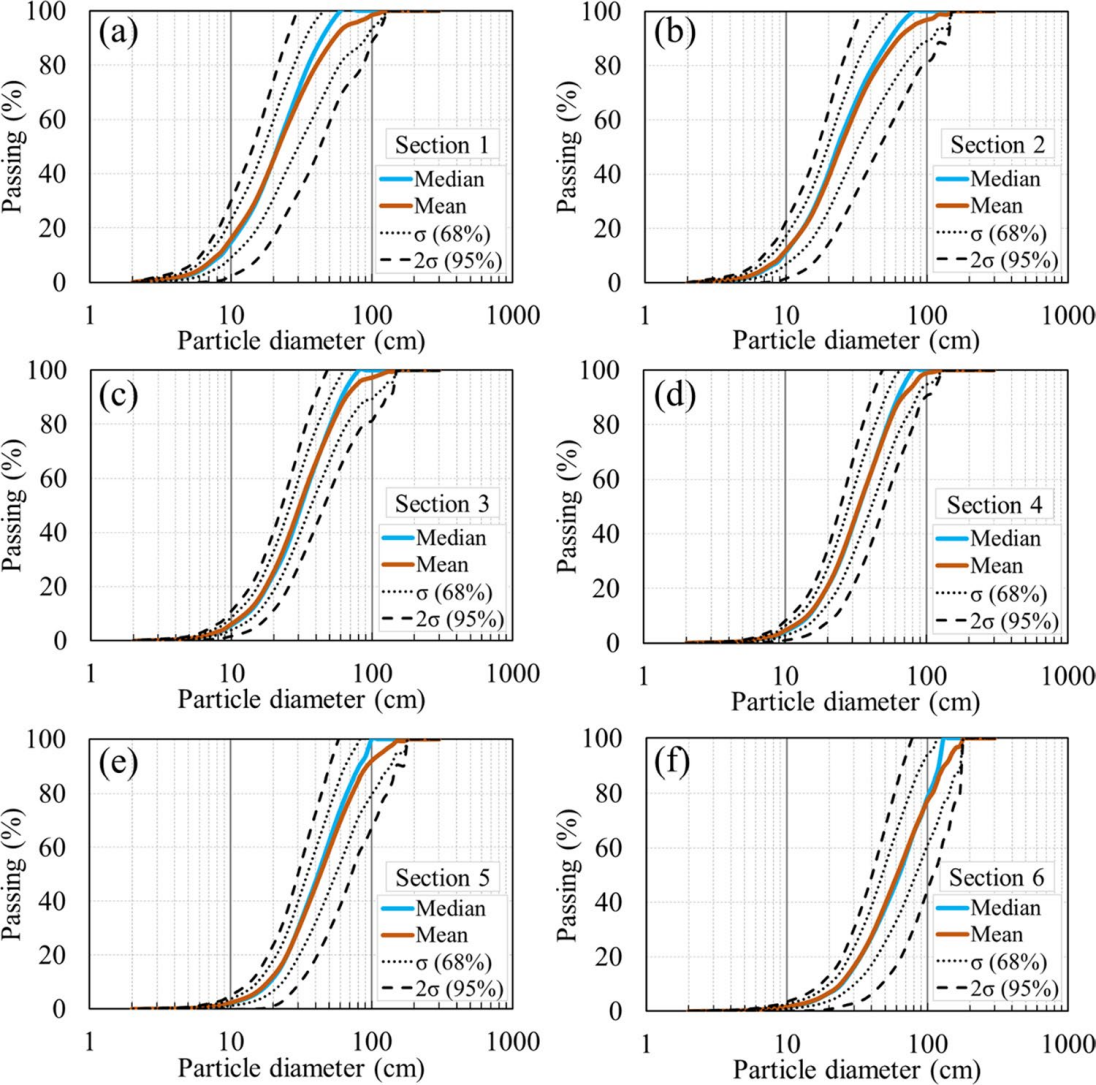
Lateral variabilities of waste rock PSD curves along the slope from **a** Section 1 to **f** Section 6. 68% envelope (dotted line) represented PSD curves with passing percentage less than one standard deviation (σ) around the mean. 95% envelope (dashed line) represented PSD curves with passing percentage less than two standard deviations of the mean

PSD curves of waste rock exhibited high lateral variability, especially for sections 1 and 6. For example, the maximum standard deviation (σ) of the passing percentage was around 17% for a particle diameter of 40 cm in Sect. “Introduction” and 18% for a particle diameter of 80 cm in Sect. 6. This indicated that in practice, particles ranging between 40 and 80 cm exhibited the highest variability along the lateral direction. The highest variability in sections 1–6 occurred at particle diameters ranging between 40 and 60 cm, with the maximum standard deviation (σ) of the passing percentage around 10%. The maximum diameter (*D*_max_) in the top section varied between 30 and 130 cm (Fig. 8a). Similar variations of *D*_max_ in sections 1–6 were also observed, ranging between 80 and 180 cm (Fig. 8b–f).

**Fig. 9:**
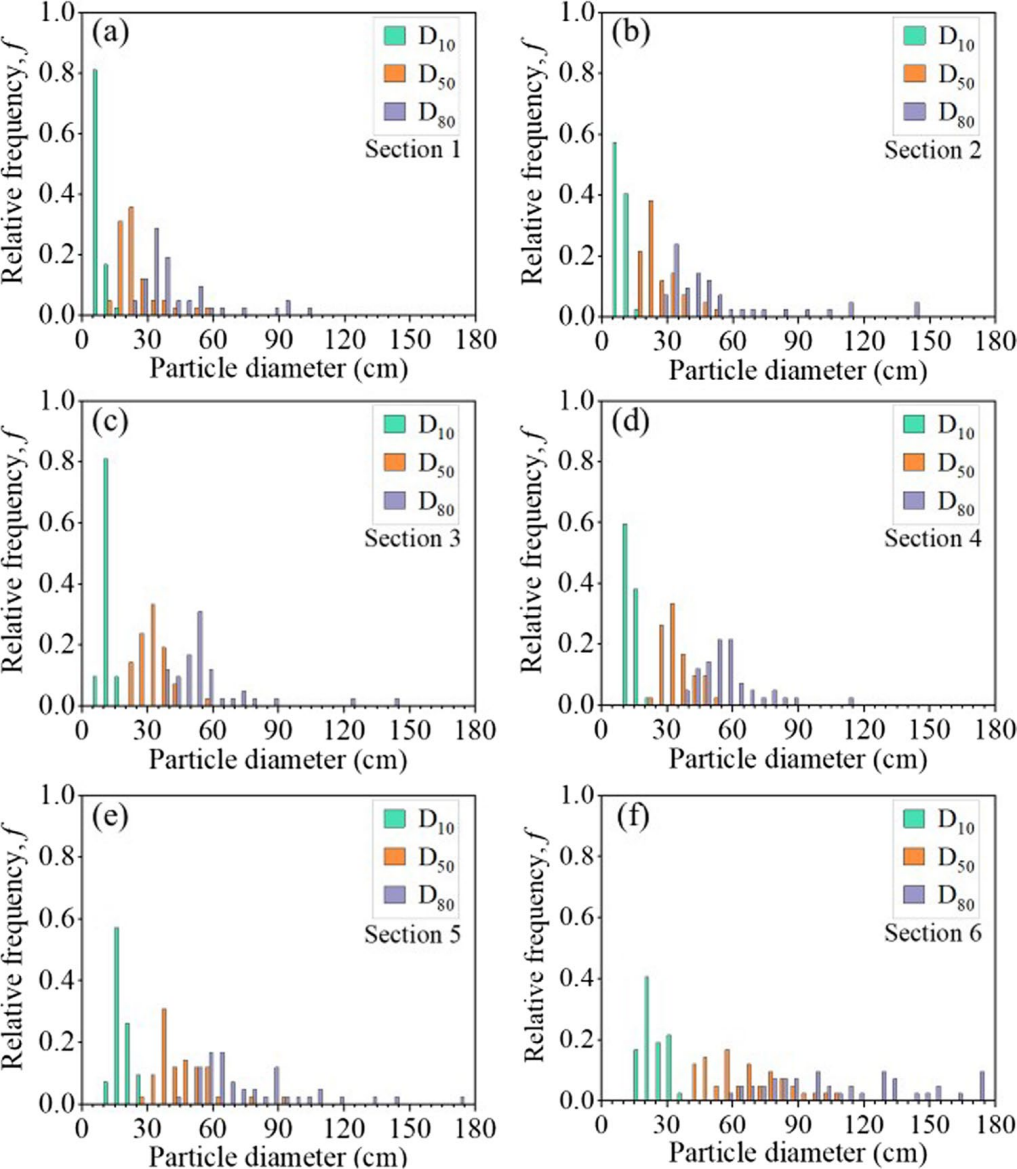
Lateral variabilities of characteristic diameters *D*_10_, *D*_50_, *D*_80_ along the slope from **a** Section 1 to **f** Section 6. The distributions of *D*_10_, *D*_50_, *D*_80_ were analyzed based on the 42 PSD curves which covered a horizontal length of 1400 m

**Fig. 10:**
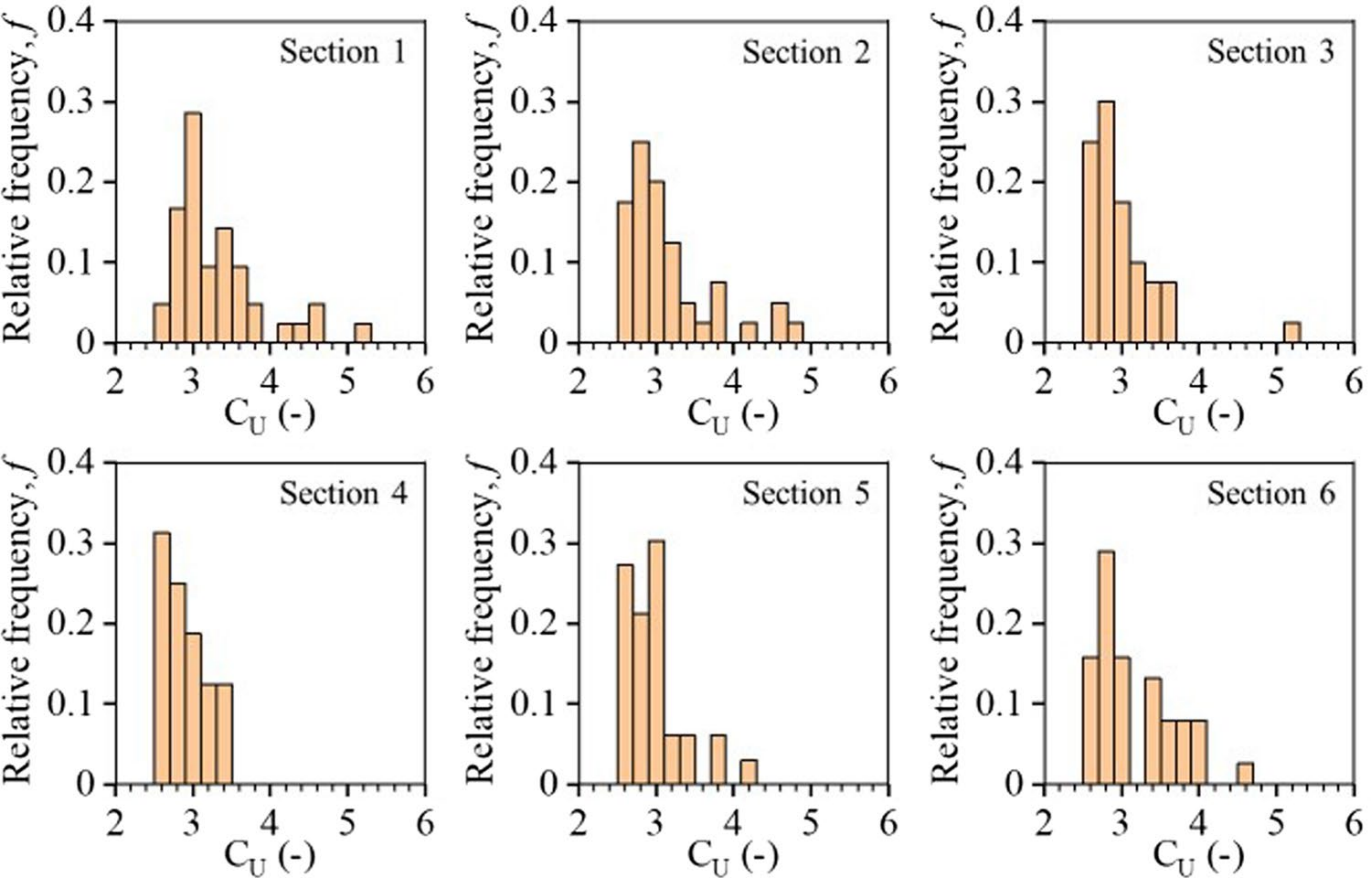
Lateral variabilities of C_U_ in the six sections. The distribution of C_U_ in each section was analyzed based on the 42 PSD curves which covered a horizontal length of 1400 m

The distributions of *D*_10_, *D*_50_ and *D*_80_ and C_U_ in each section were exhibited using the relative frequency (*f*) (Figs. 9 and 10). The maximum frequency (*f*_*m*_) decreased from *D*_10_ to *D*_80_, indicating that the lateral variation increased with particle diameters. For example, diameter *D*_10_ in sections 1 varied between 5 and 15 cm, with a maximum frequency *f*_*m*_ = 0.8 for a particle diameter of 8 cm. Diameter D_50_ was between 15 and 25 cm, with *f*_*m*_ = 0.36 for a particle diameter of 23 cm. Finally, diameter D_80_ distributed along a wider range comprised between 30 and 55 cm, with *f*_*m*_ = 0.29 for a particle diameter of 33 cm. C_U_ mainly varied between 2.4 and 5 in the 6 sections and exhibited higher C_U_ in sections 1 with *f*_*m*_ = 0.29 for a C_U_ of 3 (Fig. 10).

Similar results were also observed in other sections, both close to the top (finer zones) and the bottom (coarser zones) of the slope (Fig. 9). For example, diameter D_50_ in sections 1 was concentrated between 18 cm (*f* = 0.31) and 23 cm (*f* = 0.36), while D_50_ in Sect. 6 was between 43 and 68 cm (*f* = 0.45). Diameter D_80_ in Sect. 6 exhibited much higher variations than that in sections 1 with a maximum frequency of *f*_*m*_ = 0.1, indicating that larger waste rock particles tended to distribute more variably at the bottom of the slope.

## Result analysis and discussion

### Segregation and lateral heterogeneity in a waste rock pile

The results of the waste rock pile image analysis presented above indicated a significant vertical segregation along the slope of the 10 m high bench, with a segregation degree increasing from − 0.77 ± 0.39 in sections 1 (top) to + 0.4 ± 0.14 in Sect. 6 (bottom). Maximum particle size and characteristic diameters also tended to increase from sections 1–6. For example, D50 increased from 24 cm ± 9.5 cm in Section 1 to 66 cm ± 18 cm in Section 6 (an increase by 168%). Similarly, other waste rock piles with different heights also exhibited segregation. For example, diameter *D*_50_ at 15 m high waste rock pile (Chi 2011) increased from 21 cm in the top section to 41 cm in the bottom section, i.e., an increase by about 95%, Also, diameter *D*_50_ at an around 20 m high waste rock pile (Zhang et al. 2017) was 40 cm in the top section and 143 cm in the bottom section, i.e., an increase by about 258%.

High variations of PSD curves and characteristic diameters of waste rock along the lateral direction indicated significant heterogeneity in the studied pile. For example, diameter *D*_50_ of the original material (before the deposition) varied between 33 and 63 cm along the 1400 m of the investigated bench, with a maximum frequency of only 0.24. Similar results were also observed from other waste rock piles. For example, diameter *D*_50_ of the original waste rock (before the deposition) varied between 6 and 36 cm along the lateral direction of the 15 m high waste rock pile at Diavik mine (Chi 2011; Barsi 2017). Such lateral heterogeneity is mainly attributed to lithological and mineralogical characteristics of the host rock, as well as blast pattern and energy which affect the degree of rock fragmentation (Raymond et al. 2021; Kinyua et al. 2022).

Vertical and lateral heterogeneity is, therefore, particularly critical when determining and simulating waste rock piles geotechnical, hydrogeological, and geochemical behaviour (St-Arnault et al. 2020; Vriens et al. 2019). For example, the difference of friction angle in different locations of a pile could exceed 14° because of spatial variations of particle sizes (Zevgolis 2018; Rahmani et al. 2021) and local wetting front velocities can vary over four orders of magnitude (Nichol et al. 2005; Webb et al. 2008). The following sections, therefore, discuss various approaches to predict and account for segregation and heterogeneity in large-scale waste rock piles.

### Prediction of segregation and segregation degree

The uniformity coefficient C_U_ (= *D*_60_/*D*_10_) is often considered a practical and easy-to-use criterion to estimate possible segregation of a coarse material, and a greater *C*_U_ (usually *C*_U_ > 3) is generally deemed to indicate a higher segregation risk (Langroudi et al. 2015). However, this study has shown that such criterion should be considered carefully and may sometimes underestimate the segregation risk. Indeed, C_U_ of the original waste rock in this study was sometimes as small as 2.8, but segregation was still significant with segregation degrees around − 1.53 in sections 1 and 0.34 in Sect. 6. One of the reasons for the poor fit of the C_U_ criterion is that particles coarser than *D*_60_ are more prone to segregate (Sherard et al. 1984). For example, waste rock particles larger than 150 cm (while *D*_60_ = 50 cm) were all accumulated in the bottom section of the investigated waste rock pile. Therefore, using *D*_60_ and *D*_10_ may be not always reliable to predict segregation in the field.

**Fig. 11:**
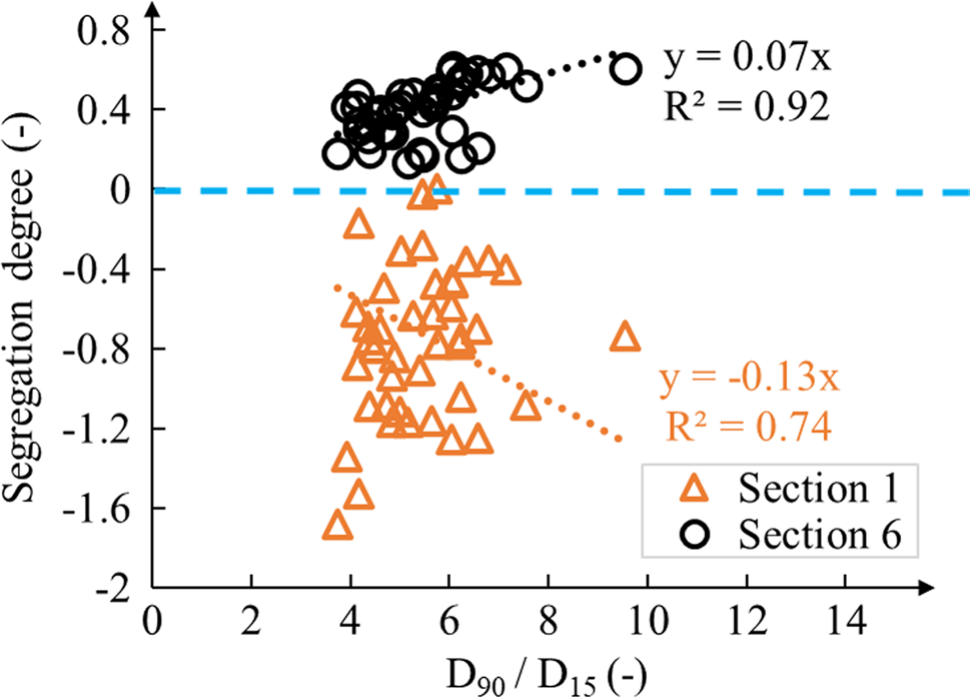
Segregation degree as a function of D_90_/D_15_ of the original waste rock in sections 1–6. A segregation degree less than 0 indicates finer material than the original and a segregation degree greater than 0 indicates a coarser material than the original

Predicting the segregation degree using greater characteristic diameters such as *D*_90_ was, therefore, considered, and a ratio *D*_90_/*D*_15_ was proposed to better evaluate segregation (Burenkova 1993; Asmaei et al. 2018). In this study, *D*_90_/*D*_15_ of the original waste rock was between 3.7 and 9.5, with an average of 6.7 ± 1.6. The segregation degree was fitted as a function of *D*_90_/*D*_15_ with an intercept of 0 considering the reality that *D*_90_/*D*_15_ is always a positive value (> 1) and waste rock tends to be homogenous when *D*_90_/*D*_15_ closes to 1. The segregation degree in Sect. 6 tended to indicate a linear relationship with *D*_90_/*D*_15_ with a coefficient of determination (*R*^2^) greater than 0.9 (Fig. 11). The fitted trend indicated that the segregation degree in the coarse zone (e.g., Sect. 6) tended to linearly increase with *D*_90_/*D*_15_, and that waste rock tended to be finer in the top of the pile (sections 1) with increasing *D*_90_/*D*_15_. However, the relation between the segregation degree and the ratio *D*_90_/*D*_15_ was less clear in the top section of the pile (*R*^2^ = 0.74) than in the bottom (*R*^2^ = 0.92), most probably because large particles can strongly affect the PSD curve in this area (similar to what is observed in Figs. 6, 7, and 8). Other researchers (Asmaei et al. 2018) also reported that the segregation degree of the whole slope tended to increase with the ratio *D*_90_/*D*_15_. However, the slope of the fitted linear relation in their study was 0.29 (*R*^2^ = 0.99), that is around 5 times greater than the fitted slope of 0.07 (*R*^2^ = 0.92) in this study. A possible reason for this difference may be that *D*_90_/D_15_ in the present study varied in a relatively smaller range (i.e., between 3.7 and 9.5) compared to their analysis (i.e., between 13 and 64). In other words, if the general trend between segregation and *D*_90_/*D*_15_ was verified in several waste rock piles, the linear relation parameter may depend on waste rock properties.

### Correction of particle size distribution for fine particles

In this study, the uniformity coefficient C_U_ in the 42 waste rock slopes ranged between 2.8 and 4.1. Similar results were obtained using image analysis in a different mine waste rock pile, where C_U_ of the whole slope was around 4.8 (Zhang et al. 2017). However, in practice, waste rock C_U_ is typically around 20 or more (Aubertin 2013). This difference was mainly attributed to the fact that particles smaller than 5 cm (i.e., correspond ding to around 25 cm^2^) were not detectable using image analysis. This is a general limitation to the technique and similar results were also reported for other waste rock piles, where the minimum particle diameter that could be detected using image analysis was between 1 and 20 cm (Chi 2011; Zhang et al. 2017).

Fine particles can, however, have a significant impact on hydrogeological (Bao et al. 2020; Essayad 2021), geotechnical (Laverdière et al. 2022) and even geochemical (Neuner et al. 2013) properties of waste rock. Therefore, the PSD curve determined using image analysis should often be corrected using field and/or laboratory measured PSD curves. These are, however, also incomplete and rarely contain particles coarser than 50 cm (Cash 2014; Barsi 2017), but there is still a rather significant overlap (2–50 cm) between directly measured and image-based PSD curves so they can be combined.

In this study, around 1000 kg of waste rock were sampled at Canadian Malartic mine for PSD analysis (Essayad 2021). Waste rock particles larger than 10 cm and up to 25.4 cm were directly and manually characterized in the field (particles coarser than 25.4 cm were not characterized for practical reasons) and waste rock smaller than 10 cm was transported to the laboratory for sieve analysis (diameter > 0.075 mm) and hydrometer tests (diameter < 0.075 mm).

**Fig. 12:**
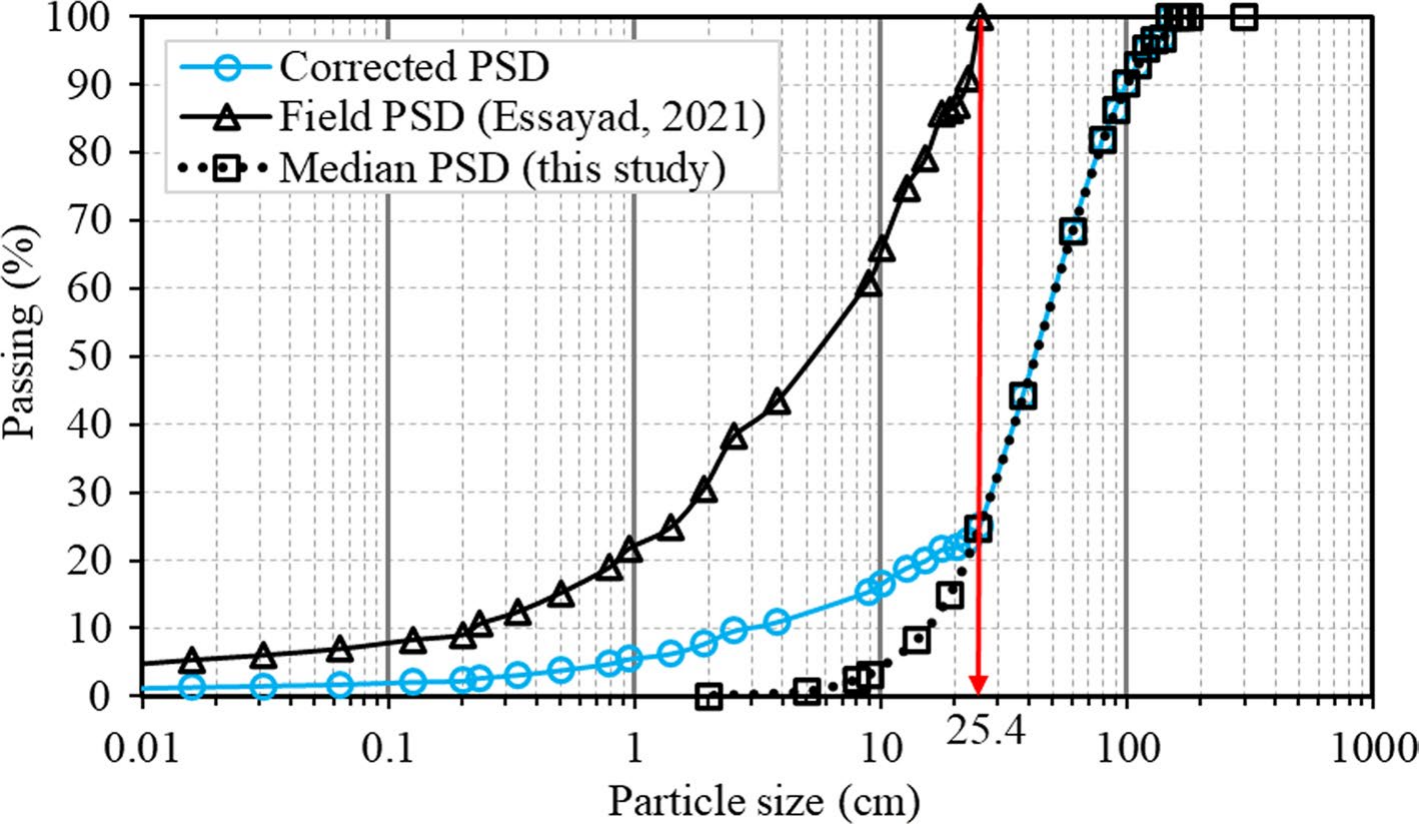
Correction of waste rock PSD curve at Canadian Malartic mine (in blue) by integrating field measured PSD (between 0 and 25.4 cm; black triangles) and image analysis (between 25.4 cm and 150 cm; black squares)

The median PSD curve (Figs. 6a and 12) of the original waste rock obtained from image analysis was corrected and combined with measured PSD curve. In the corrected PSD curve, the passing percentages of fractions larger than 25.4 cm were kept the same to those in the median PSD curve from image analysis. The correction was mainly focused on size fractions smaller than 25.4 cm, which accounted for 25% of the median PSD curve of image analysis sample. The corrected passing percentage of fractions smaller than 25.4 cm were then calculated through multiplying the passing percentage of each diameter in measured PSD curve by 25%. These recalculated PSD fractions (smaller than 25.4 cm) were finally combined with the median PSD fractions larger than 25.4 cm. A similar correction approach was used to correct PSD curves by integrating measured and image analysis obtained PSD curves in other studies (Cash 2014; Zhang et al. 2017). The resulting PSD curve covered particle diameters from 10^–4^ cm (measured in the laboratory) to a maximum diameter of 150 cm (estimated using image analysis). The corrected *D*_10_ (= 2.9 cm) was 1/5 of that in the original median PSD curve determined from image analysis only (Fig. 12), resulting in a *C*_U_ = 18 (compared to *C*_U_ = 3.4 from image analysis). The correction of field PSD curve was made for the waste rock of the whole slope, and this corrected PSD curve was used to characterize waste rock in Canadian Malartic mine. Such correction can have a significant impact on the estimation of waste rock properties, but assumes that this correction process can be applied in segregated sections, although fractions smaller than 25.4 cm in these segregated sections would be slightly different (see the next section below).

### Effect of segregation and lateral heterogeneity on waste rock pile properties

#### *Effect of segregation and lateral heterogeneity on the estimation of *in situ* saturated hydraulic conductivity*

In practice, vertical segregation and lateral heterogeneity have a direct impact on waste rock hydrogeotechnical properties. For example, the variations of saturated hydraulic conductivity (*k*_sat_) could affect the hydrogeotechnical properties and result in preferential flow within waste rock piles (Fala, et al. 2005, 2012). Sometimes, one or two orders of magnitude difference in the hydraulic conductivity can make water flow along irregular and diverted paths (Lahmira et al. 2016, 2017), thus challenging waste rock reclamation. In this study, the effect of segregation and lateral heterogeneity on waste rock hydrogeological behaviour was investigated using Kozeny–Carman-Modified (KCM) model (Eq. 6; Mbonimpa et al. 2002) and Taylor model (Eq. 7; Taylor 1948), which are both regularly used to predict waste rock saturated hydraulic conductivity (Peregoedoa et al. 2013; Bréard Lanoix et al. 2020 et al. 2020; Essayad 2021).

KCM model (Mbonimpa et al. 2002):

6 $$k_{{{\text{sat}}}} \left( {\frac{{{\text{cm}}}}{s}} \right) = C_{{\text{G}}} \frac{{\gamma_{{\text{w}}} }}{{\mu_{{\text{w}}} }}\frac{{e^{(3 + x)} }}{(1 + e)}C_{{\text{U}}}^{1/3} D_{10}^{2}$$

where *C*_G_ is a constant (*C*_G_ = 0.1 for granular material); *γ*_w_ is unit weight of water (*γ*_w_ = 9.8 kN/m^3^); ${\upmu }_{{\text{w}}}$ is dynamic viscosity of water (≈ 10^–3^ N.s/m^2^ at 20℃); e is void ratio [-]; x is tortuosity factor (*x* = 2 for granular material); *C*_U_ is uniformity coefficient [-]; D_10_ is particle diameter corresponding to 10% passing (cm).

Taylor model (Taylor 1948):

7 $$k_{{{\text{sat}}}} \left( {\frac{{{\text{cm}}}}{s}} \right) = C_{1} \frac{{\gamma_{{\text{w}}} }}{{\mu_{{\text{w}}} }}\frac{{e^{3} }}{(1 + e)}D_{50}^{1.5}$$

where *C*_1_ is a shape factor (*C*_1_ = 0.004 is suggested for waste rock by Peregoedoa et al. (2013)); $\gamma_{{\text{w}}}$ is water unit weight (= 9.8 kN/m^3^); $\mu_{{\text{w}}}$ is dynamic viscosity of water (≈ 10^–3^ N.s/m^2^ at 20 ℃); e is void ratio [–]; D_50_ is particle diameter corresponding to 50% passing (cm).

**Fig. 13:**
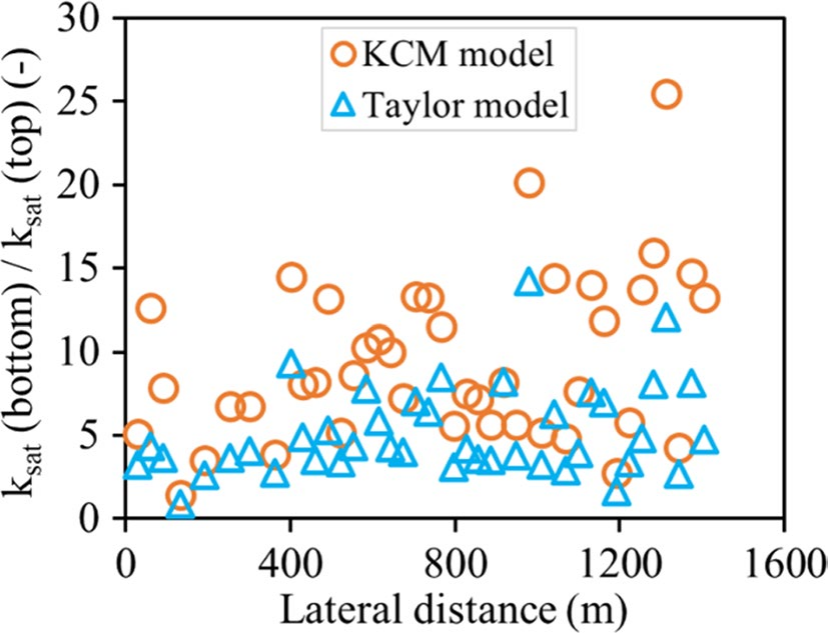
Variation of ratio *k*_sat_ (bottom)/*k*_sat_ (top) as a function of lateral heterogeneity. Waste rock saturated hydraulic conductivity *k*_sat_ was predicted using KCM (Eq. 6) and Taylor (Eq. 7) models

The ratio of waste rock saturated hydraulic conductivity in the bottom section divided by that in the top section, i.e., *k*_sat_ (bottom)/*k*_sat_ (top), varied between 1.4 and 25.5 with KCM model and between 0.9 and 14.3 with Taylor model (Fig. 13). In other words, waste rock segregation during deposition could induce vertical variations of hydraulic conductivity by around one order of magnitude in Canadian Malartic waste rock pile. Similar trends were also observed in other waste rock piles, where the permeability in the bottom section was 3–12 times greater than that in the top section (Zhang et al. 2017; Chi 2011). This effect could even be more pronounced because of the crushing and compaction of waste rock at the surface of the pile because of the frequent circulation of haul trucks (Fala et al. 2012; Maknoon et al. 2021; Raymond et al. 2021).

Lateral heterogeneity of waste rock particle sizes also induced significant variations of saturated hydraulic conductivities. No clear trend was observed for the lateral distribution of ratios *k*_sat_ (bottom)/*k*_sat_ (top) which were randomly distributed and could exceed 16 (Fig. 13). Such field results are scarce in the literature, but similar lateral variations (up to 25 times) were also observed for saturated hydraulic conductivity of waste rock samples measured in the laboratory (Cash 2014).

These results tend to confirm the representativity of numerical simulations which consider heterogeneity by randomly distributing materials with differe0nt permeabilities (with one order of magnitude difference) within the whole waste rock pile (Lahmira et al. 2016, 2017). However, this study has also shown that waste rock sizes exhibited a strong vertical correlation, which could be considered to improve the way to simulate segregation in waste rock piles.

Here, the discussion was conducted in terms of saturated hydraulic conductivity, but the effect of segregation on waste rock shear strength (Kim et al. 2014; Zevgolis 2018) and geochemical reactivity (St-Arnault et al. 2020; Seigneur et al. 2021) is expected to be similar.

#### Comparison of original non-segregated and segregated waste rock saturated hydraulic conductivity

The saturated hydraulic conductivity (*k*_sat_) is usually determined by characterizing waste rock samples (before the deposition, i.e., non-segregated) in the laboratory and as the measured result is deemed representative of field waste rock (Abdelghani et al. 2015; Maknoon et al. 2021). However, the analyses above indicated that segregation could significantly affect in situ hydraulic conductivity. The saturated hydraulic conductivity was predicted using KCM (Eq. 6) and Taylor (Eq. 7) models, but this time based on the PSD curves of each section corrected using the approach proposed above and including fine particles (i.e., diameter < 5 cm). The void ratio *e* = 0.3 was determined from field measurements at Canadian Malartic mine (Essayad 2021).

**Fig. 14:**
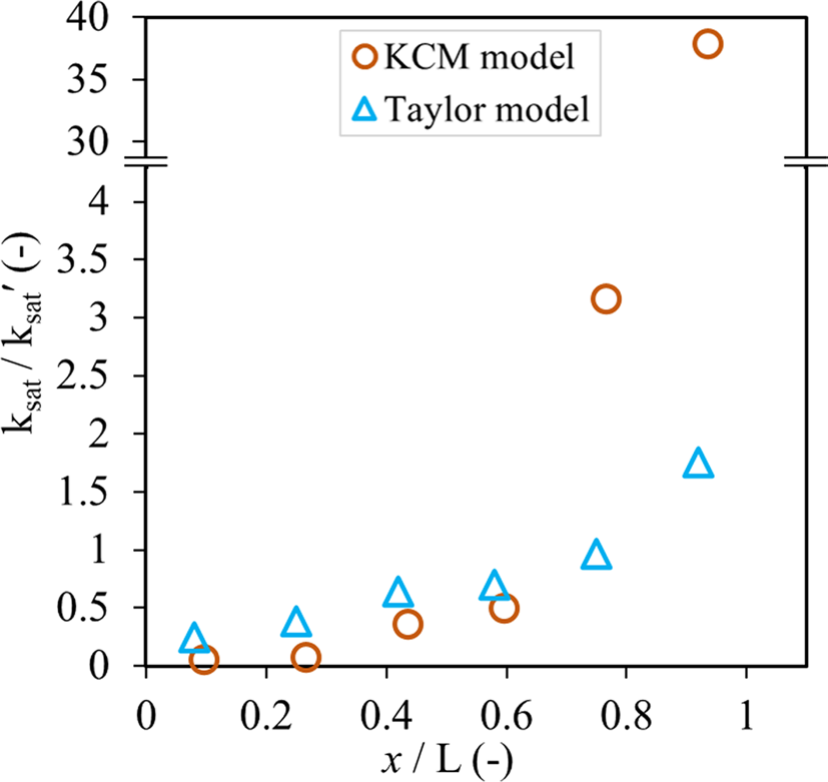
Variation of ratio *k*_sat_/*k*_sat_ʹ as a function of vertical positions along the slope. Waste rock saturated hydraulic conductivity k_sat_ was predicted using KCM (Eq. 6) and Taylor (Eq. 7) models

The ratio of waste rock saturated hydraulic conductivity (*k*_sat_) in each section divided by the saturated hydraulic conductivity of original waste rock, i.e., *k*_sat_/*k*_sat_ʹ, was 0.06 in sections 1 (*x*/*L* = 0.08, top of the slope) with KCM model and 0.3 with Taylor model (Fig. 14). The ratio *k*_sat_/*k*_sat_ʹ in Sect. 6 (*x*/*L* = 0.92, bottom of the slope) was 38 with KCM model and 1.8 with Taylor model. The saturated hydraulic conductivity in the middle of the slope (*x*/*L* = 0.6 ~ 0.8) was similar to that of the original waste rock (*k*_sat_/*k*_sat_ʹ ≈ 1). In other words, the saturated hydraulic conductivity of the original unsegregated waste rock could be one order of magnitude greater than that in the top of the pile and one order of magnitude smaller than that in the bottom of the pile. The saturated hydraulic conductivity of the original waste rock is, therefore, not representative of the waste rock properties in the slope (as also suggest by Wilson et al. 2013 and Barsi 2017) and can in practice vary by more than one order of magnitude for a 10 m high bench. In practice, the saturated hydraulic conductivity of waste rock tended to be overestimated in the top section and underestimated in the bottom section when this correction method was used to correct the waste rock from image analysis.

### Particle shape effect

In this study, particle diameters and masses were determined using ellipsoid model, and the short axis of the particles was estimated using a shape factor, a = c/b = 0.53. This shape factor was, however, measured in the laboratory and assumed constant and independent of the particle diameter, while in practice, it may decrease with particle diameter (Linero et al. 2017). Even though the field shape factor could not be determined because of the lack of data, the influence of choosing a constant value was investigated by comparing the shape of around 10,000 particles in the field (using image analysis) with the results of the 2200 particles analyzed in the laboratory (see methodology section).

**Fig. 15:**
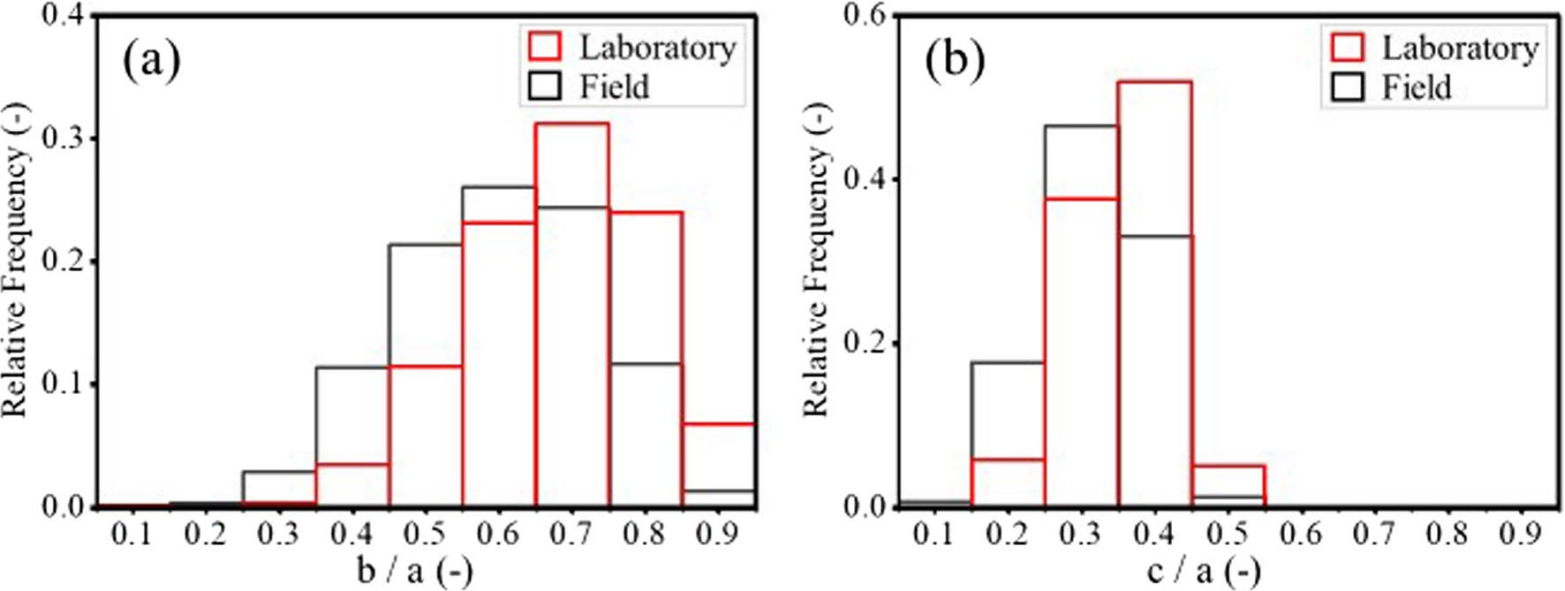
Waste rock particle shape ratios **a** *b*/*a* and **b** *c*/*a* in the laboratory (red) and in the field (black). a, b and c are the long, medial and short axis of waste rock particles. The comparison is based on the analysis of 2224 particles in the laboratory with diameters between 0.8 cm and 3.8 cm, and 10 000 particles in the field with diameters larger than 10 cm (assuming a constant shape factor *c*/*b*)

The ratio *b*/*a* of waste rock varied between 0.5 and 0.8 in the laboratory and between 0.4 and 0.8 in the field (Fig. 15). The ratio *c*/*a* ranged between 0.3 and 0.4 in the laboratory and between 0.2 and 0.4 in the field. Although waste rock generally exhibited similar shapes in the laboratory and in the field, the distribution of both *b*/*a* and *c*/*a* tended to be slightly wider in the field than in the laboratory. For example, the maximum frequency of *b*/*a* was 0.26 in the field, i.e., slightly less than the 0.31 measured in the laboratory. One reason for this discrepancy can be that significantly more particles were analyzed in the field, thus statistically covering a larger variety of shapes. Another reason could be that ratios *b*/*a* (Linero et al. 2017) and *c*/*a* (Ovalle, et al. 2020) both tended to increase with particle diameters.

**Fig. 16:**
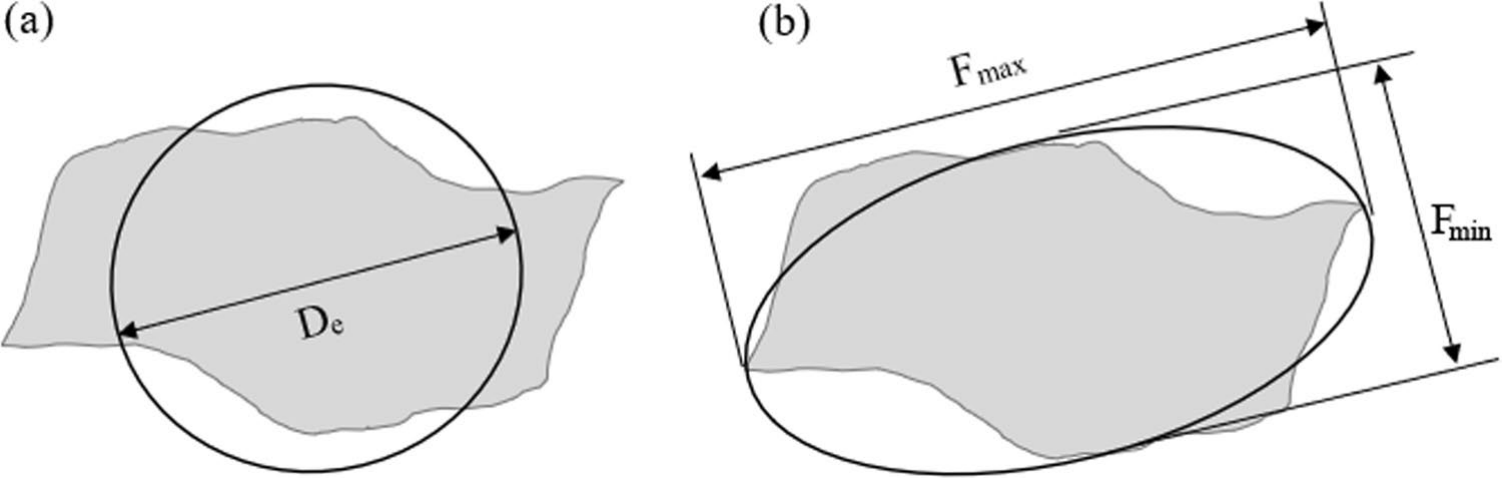
Shape characterization using **a** sphere model and **b** Feret’s diameter model. D_e_ represents the equivalent particle diameter; *F*_max_ and *F*_min_ represent the maximum and minimum Feret’s diameters

The ellipsoid model (Fig. 3) is usually considered efficient to estimate the dimensions of granular particles, but, as mentioned above, the estimation of the short axis can be difficult. Other models such as sphere model and Feret’s diameter model were, therefore, also used in this study to determine waste rock PSD. Sphere model (Fig. 16a) is easy to apply when the specimen comprises a large number of particles (Shanthi et al. 2014; Zhang et al. 2017). Measured particle area (A) from image analysis is converted to an equivalent particle diameter (*D*_e_, $D_{{\text{e}}} { } = 2 \times \sqrt {A / \pi }$), which is then used to calculate sphere volumes and determine their mass (Li et al. 2005). Feret’s diameter model (Fig. 16b) is also an ellipsoid-based shape model except that the long and medial axes are defined by maximum (*F*_max_, the longest distance between any two points along the particle boundary) and minimum (*F*_min_, the minimum caliper diameter of the particle) Feret’s diameters (Ferreira and Rasband 2012; Hamzeloo et al. 2014). The equivalent particle diameter is then calculated as $D_{{\text{e}}} = F_{\min } / \sqrt 2$.

**Fig. 17:**
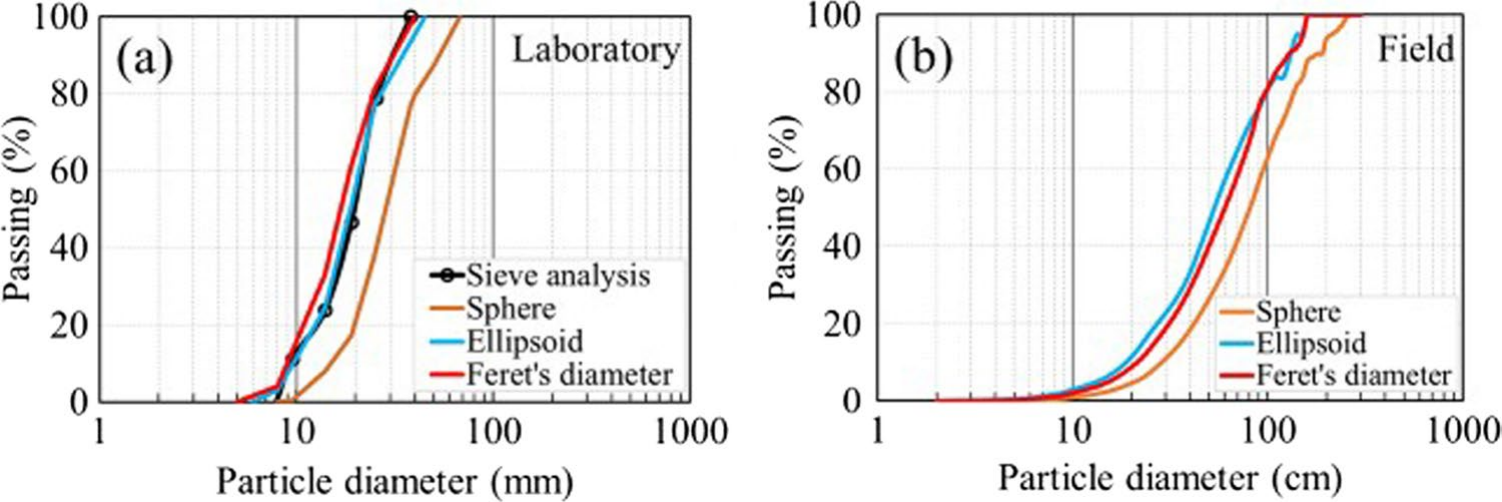
PSD curves of waste rock in the **a** laboratory and **b** field using sphere, ellipsoid and Feret’s diameter models in image analysis. The PSD curve determined with sieve analysis in the laboratory was used for comparison

Among these three models, the PSD curve obtained using the ellipsoid model was the closest to the PSD curve measured using sieve analysis in the laboratory (Fig. 17a). At field scale (Fig. 17b), Feret’s diameter model results were similar to ellipsoid model. However, PSD obtained using sphere model was always coarser than that using ellipsoid model and Feret’s diameter model both in the laboratory and in the field (Fig. 17). The main reason is that waste rock particles are mostly elongated and using a sphere, therefore, tends to overestimate the diameter under the same equivalent projected area. From the comparison above, ellipsoid model is considered the best to estimate waste rock shapes in general, but Feret’s diameter model can also give good results in the field.

### Discussion

Image analysis was used in this study to quantify both segregation and lateral heterogeneity in a waste rock pile. Despite its numerous advantages (e.g., easy operation, low cost, high efficiency), image analysis also has a few limitations, and several assumptions were made to interpret the results. First, the boundary of every single particle was detected based on pixel intensity differences between the particles and the background. However, pixel intensity is sensitive to image brightness and resolution (Ferreira and Rasband 2012; Đuriš et al. 2016; Zhang et al. 2017), and detection of particle boundaries may be difficult if pixel intensity is not contrasted enough, resulting in an overestimation particle diameter. Second, different colours within the same particle (for example, because of different mineral composition) or brightness (because of shades), may lead the image processing code to distinguish several smaller particles, where there is just on (Azzali et al. 2011). Particle overlap would hide parts of the particles and also result in underestimation of these particle (Zhang 2016). In addition, the pixel conversion factor was 1.2 cm in this study, and particles smaller than 2 cm could not be detected, even though they represented (based on direct PSD measurement) about 8% of the entire PSD curve. Neglecting fine particles could have a significant impact on the estimation of waste rock hydrogeotechnical properties (Chapuis 2012). Higher resolution cameras are, therefore, recommended to improve the precision of particle detection. Higher resolution cameras and/or lower flight altitude are, therefore, recommended to improve the precision of particle detection. For example, the resolution of recent cameras can be greater than 2.7 mm/pixel for large-scale detection (Westfeld et al. 2015; Chabot et al. 2016; Di Felice et al. 2018; Inzerillo et al. 2022). Other remote sensing techniques such as LiDAR could also be an alternative option (Collin et al. 2018; Li et al. 2020).

This study focused on the segregation and lateral heterogeneity on the surface of a waste rock pile. However, internal heterogeneity can also be significant because of the presence of the alternance of inclined fine- and coarse-grained layers (Anterrieu et al. 2010; Bao et al. 2020). Such internal structure is, however, difficult to characterize using only image analysis and would require additional investigation methods, such as geophysical methods (Anterrieu et al. 2010; Azzali et al. 2011; Dimech et al. 2019), trench excavation (Stockwell et al. 2006; Boekhout et al. 2015) and numerical simulations (Raymond et al. 2021; Qiu et al. 2022). In addition, lateral heterogeneity was characterized along the slope of a field waste rock pile, but no clear trend could be observed along the lateral direction. More field observations on different waste rock piles are recommended to improve the characterization of lateral heterogeneity using different techniques, such as field excavation (Essayad 2021), in-situ infiltration test (Lanoix et al. 2020) or electrical resistivity tomography (ERT) (Binley et al. 2015; Dimech et al. 2019). Field excavations in particular would contribute to directly and precisely determine PSD curves for various sections and also depths (which could not be investigated using image analysis). Infiltration tests and ERT investigations are indirect techniques that would also give an overview of waste rock PSD distribution with depth.

Finally, this study was carried out only for a 10 m bench of waste rock pile at Canadian Malartic mine which exhibits similar mineral composition. Even though comparison with literature has shown similar trends in other waste rock piles, segregation analyses on more waste rock piles are recommended to confirm the field observations and contribute to further understand heterogeneity of waste rock piles.

## Conclusions

In this study, waste rock segregation and lateral heterogeneity were characterized using image analysis on drone pictures obtained from Canadian Malartic mine, a surface gold mine located in Quebec, Canada. A total of 42 drone images, covering an area with 1400 m wide and 10 m high (one bench), were analyzed. Ellipsoid model was used to represent the irregular shapes and a calibrated shape factor of 0.53 was applied to determine waste rock diameters and masses. The bench slope was divided into six sections in the vertical direction and the PSD curve in each section was determined. A total of 252 PSD curves were analyzed to quantify waste rock segregation and heterogeneity. The segregation degree and the characteristic diameters from the top to the bottom section of the slope were obtained and quantitatively compared. Most important findings can be summarized as follows:

1. The increase of relative particle diameters and segregation degree indicated significant segregation along the slope of waste rock pile. Segregation degree increased from − 0.77 ± 0.39 in the top (finer zone) to + 0.4 ± 0.14 in the bottom (coarser zone). The strong vertical correlation of waste rock sizes characterized in this study could improve the way to simulate segregation in waste rock piles.
2. Significant lateral heterogeneity was characterized by investigating the lateral distribution of waste rock particles. The maximum diameters varied between 30 and 130 cm in the top section and between 80 and 180 cm in the bottom section. The maximum frequency of characteristic diameters *D*_50_ and *D*_80_ were always smaller than 0.4.
3. Segregation and lateral heterogeneity resulted in high variations (one to two orders of magnitude difference) of the saturated hydraulic conductivity and more generally of the geotechnical properties of waste rock in the field. The saturated hydraulic conductivity in the bottom section was up to 26 times than that in the top section.
4. Image analysis with ellipsoid model is an efficient method to characterize waste rock segregation and lateral heterogeneity in large-scale waste rock piles. A calibrated constant shape factor a = 0.53 can well estimate the short axis of waste rock particles in this study. The image resolution constrained particle detection precision, and fine particles (e.g., < 5 cm) were, therefore, underestimated in image analysis.

This research should provide a reference to quantitatively characterize waste rock segregation and lateral heterogeneity in the field and provide the basis to investigate segregation in numerical simulations in future works.

## Data Availability

The data presented in this study are available on request from the first or corresponding authors.
